# The Effect of a Tailored Health Education Programme on Medication Management in the Elderly

**DOI:** 10.1155/2020/1903191

**Published:** 2020-05-13

**Authors:** Salma Mohamed Samir El Said, Ghada Essam El-Din Amin, Essam Mohamed Baumy Helal, Reham Salah Amin Radwan, Hoda MF Wahba

**Affiliations:** ^1^Geriatrics and Gerontology Department, Ain Shams University, Cairo, Egypt; ^2^Community, Environment and Occupational Medicine Department, Ain Shams University, Cairo, Egypt; ^3^General Internal Medicine Department, Ain Shams University, Cairo, Egypt; ^4^Family Medicine Department, Ain Shams University, Cairo, Egypt

## Abstract

**Background:**

Empowering the elderly by education programs can decrease medication problems, morbidity, and mortality.

**Methods:**

A cross-sectional study to identify trends and baseline medication management among the elderly in nursing homes followed by an interventional study (tailored educational programme) offered within the same population followed by reassessment of the same medication management domains.

**Results:**

There was no effect regarding nursing home participants' medication knowledge before and after intervention, while there were variable degrees of significant statistical differences in how the participants obtain and take their medications as well as their total deficiency scores before and after intervention. Other domains were also variably affected.

**Conclusion:**

It is vital to ensure that patients have sufficient knowledge regarding their medications and how to handle and administer them. Different domains may variably be affected by educational programmes mainly due to preassessment deficits. Educational programmes need to be tailored according to the requirements of the population targeted.

## 1. Background

The National Coordinating Council for Medication Error Reporting and Prevention (NCCMERP) defines medication error as “any preventable event that may cause or lead to inappropriate medication use or patient harm, while the medication is in the control of the healthcare professional, patient, or consumer.” [1] Medication error is classified into five stages according to the phase of the medication use process: prescribing, transcribing, dispensing, administering, and monitoring [2].

Medication errors are common and represent a major cause of harm [3]. They occur at all stages of the medication-use process, most frequently at the prescribing and administering stages. In the USA, it is estimated that medication errors harm at least 1.5 million people, kill 106,000 people, and cost at least $3.5 billion annually [4]. In Asian countries such as India, the overall incidence of medication errors was found to be 21.8% [5]. In Middle Eastern countries, where studies are relatively few in number and are of poor quality, incidence rates varied from 7.1% to 90.5% for prescribing errors and 9.4% to 80% for administering errors [6]. In Egypt, 54% of errors were prescribing errors followed by monitoring errors at 25% and administering errors at 16% [7].

Elderly patients use medications more frequently than any other age group. On average, the elderly consume three times as many medications as the nonelderly [8] and use two to six prescribed medications simultaneously [9]. In addition to high rates of medication consumption in this age group, age-related physiological changes and comorbidities that often modify medication metabolism patterns and pharmacological activity can place the elderly at significant risk for medication-related issues [10].

Burge and colleagues identified a group of factors affecting incorrect medication use and patient compliance including patient, physician, and economic variables, characteristics of medicines, and medication knowledge [11].

In hospital settings, factors differ from other settings and include individual factors and system issues [12].

Up to 59% of elderly patients make errors while taking their prescriptions, and nearly 20% of hospital admissions of the elderly are attributed to self-made errors in taking medication [13]. Therefore, the UK's National Institute for Health and Care Excellence (NICE) recommends that prescribers should “when possible, encourage patients to take responsibility for their medicines and self-manage their conditions.” [14]

According to a study by Modig and team, a large percentage of these errors are attributed to knowledge gaps [15].

It is widely shown in the literature that patient education is an essential way to enhance medication adherence [16]. A significant amount of information that is transmitted orally to patients may be forgotten. Thus, written information should be provided to reinforce the message, foster the safe use of the medication, achieve better treatment adherence, and increase patient satisfaction with the care received [16].

It has also been shown that visual aids can improve the patient's understanding. Furthermore, visual cues accompanied by oral instructions have been shown to increase the patient's recall more than oral instructions alone [17].

Therefore, since medication adherence is significantly related to patients' knowledge of and attitudes towards medication, it is necessary to assess general knowledge of medication among the elderly and gather more insight into this aspect [15, 18].

## 2. Methodology

The present study was carried out in 2 phases:

  Phase 1: cross-sectional study
  Phase 2: interventional study

The data were collected from February 2017 to February 2018. Elderly aged sixty years or older were enrolled unless they were suffering from any neuropsychological disorder affecting intellectual function (e.g., dementia) or were totally dependent on a caregiver for medication intake.

The calculated sample size of phase 1 was found to be 400 elderly, and the proportion of persons with knowledge regarding drug safety was determined to be 50% ± 5 at a CI of 95% using the Epi Info 7 program. The sample was divided into 200 participants from nursing homes and 200 participants from the community (100 participants from the outpatient geriatric clinic and 100 participants from geriatric clubs).

The calculated sample size of phase 2 was found to be 50 elderly from nursing homes, which achieved 80% power to detect an odds ratio of 3.5 with a significance level of 0.05. The odds ratio is equivalent to a difference of 30% between the proportion of persons with enough knowledge before and after the intervention programme (58% before intervention and 88% after intervention).

Given the purpose of this study, only the results of the elderly living in the nursing home will be presented.

The 200 residents enrolled in the first phase were every other name from the list of occupants at the nursing homes. Phase 2 participants were the first 50 accepting to continue the study and showing interest and determination to complete the study (to guard against dropouts).

All participants were assessed by the principal investigator in a quite private setting through the following tools:

(I) Comprehensive geriatric assessment:
  1. Full history
  2. Assessment of cognitive function (Mini-Mental State Examination; MMSE, Arabic version) [19]
  3. Assessment of mood (Geriatric Depression Scale; GDS 15, Arabic version) [20]
  4. Assessment of function (Activity of Daily Living; ADL and Instrumental Activity of Daily Living; IADL scale, Arabic version) [21]
(II) The “Medication Management Instrument for Deficiencies in the Elderly Questionnaire (MedMaIDE)” [22] translated and validated
(III) An Arabic version of the validated “Morisky, Green, and Levine (MGL) Adherence Scale” or “Medication Assessment Questionnaire (MAQ)” [23]
(IV) An Arabic version of the validated Beliefs about Medicines Questionnaire—Specific (BMQ-S) [24].
(II, III, IV supplement).

Phase 2: the health education programme was carried out in the form of two interactive lectures of one-hour duration each. The first lecture included information about medication storage, expiration date, side effects, over-the-counter medications, and self-medications, while the second lecture, which was repeated after one week, included information about herbal medications, ways to help the elderly remember their medications, the importance of following the physicians' instructions, and the rules of discontinuing their medications and obtaining refills. The elderly were invited to ask questions even if those questions were unrelated to the topics of the lectures. A number of questions were asked at the beginning of each lecture as a pretest of the general knowledge of the elderly about the topics to be discussed in the lecture, and at the end of each lecture, the same questions were asked as a posttest to check the feedback of our participants, and correct answers were shared with the audience. Then, a final evaluation was performed one week after the second lecture to evaluate the results of the health education programme.

The educational intervention tool consists of a one-page A4-size paper brochure developed specifically for this study. It is written in simple Arabic for literate participants, with nine points printed in size 23 fonts for easy reading. The back of the brochure was designed for illiterate participants and showed seven pictures depicting adherence, pill boxes, correct and incorrect medication storage, and medication expiration dates. The background of each side used three colours that were chosen to be comforting to participants. The brochure was distributed to each participant at the beginning of the educational session, and a number of copies were left for the social worker to disseminate.

### 2.1. Data Analysis

- The collected data were analysed using Statistical Package for Social Science software (IBM Corp. Released 2013. IBM SPSS Statistics for Windows, Version 22.0. Armonk, NY: IBM Corp.)
- Descriptive statistics were produced for all variables and expressed as the mean ± SD for quantitative data, whereas qualitative data were expressed as numbers and percentages
- For quantitative data, comparisons between groups' scores were performed using independent *t*-tests and paired *t*-tests
- For qualitative data, comparisons between groups' category levels were performed using the chi-square test and McNemar's test
- Associations between sociodemographic variables and knowledge, adherence, and medication beliefs were determined using the Spearman correlation test and the chi-square test
- A *p* value ≤0.05 was chosen as a level of significance and ≤0.01 was chosen as a level of high significance

## 3. Results

The first stage of the study: preintervention included two hundred elderly at six nursing homes located in Cairo. Four of the nursing homes are in the private sector, while two are in the governmental sector. The second stage: intervention and postintervention included only the first fifty participants from the first sample group. Regarding the demographic data of the whole sample, the mean age was 71.43 ± 8.5 years, 72% were females, 57.5% were widowed, and the rest married, divorced, or single; most of the females were previously housewives, while the males were formerly employees of the government; 38% had university education, 23% had secondary/technical education, and 20% were illiterate; 81% were nonsmokers; and income ranged from 1,000 to more than 5,000 L.E. among the study group.

Regarding help with medication,

1. 77.8% did not receive help
2. Regarding the type of help, 50.9% received help with obtaining medication, 36.8% received help with taking medication, and 12.3% received reminders to take their medication

Tables 1 and 2 show how the elderly keep and get their medications, respectively.

According to the nursing home residents, their most important problems were “Cannot name all of my medications” (70.8% of participants) and “Cannot identify if there is a problem after taking the medication” (61.3% of participants), and the least common was “Don't know why he/she is taking each medication” (21.8% of participants). A total of 69.5% of nursing home residents had never taken any herbal medicines.

The MedMaIDE total deficiency score items before intervention showed no significant correlation with age, gender, or social status of the participants (*p* value 0.085, 0.075, 0.282, respectively), while there was a highly significant negative correlation with educational level and monthly income of all participants (*p* value 0.000 and 0.000).

Table 3 compares MedMaIDE score items among nursing home participants before and after intervention.

There was a highly significant association between the age, gender, social status, pre-retirement occupation, educational level and monthly income of participants, and their medication adherence before intervention as assessed by MAQ (*p* value 0.000, 0.004, 0.000, 0.002, 0.000, and 0.000, respectively).

Table 4 compares MAQ categories before and after intervention among nursing home participants.

Table 5 compares MAQ items before and after intervention among nursing home participants.

The statistical analysis also revealed there was a significant negative correlation between age of participants and their beliefs about medications (*p* value 0.049), while there was no significant correlation between participants' gender, social status, and monthly income before intervention (*p* value 0.707, 0.934, 0.158, 0.266, respectively). There was also no change in medication belief scores (BMQ-S) in mean or standard deviation before intervention and after intervention in nursing home participants (34.39 ± 6.196, 34.57 ± 6.220, respectively).

## 4. Discussion

According to many studies, intervention approaches used so far have produced inconsistent effects on long-term improvement in the management of medications. The authors of this work believe there are many factors affecting this situation that differ greatly from one care setting to another. Similarly, different cultures may have a different constellation of deficits requiring a specific targeted educational programme. Educational level and culture play a very important role in the causes of noncompliance. It is therefore vital to perform studies in different populations and care settings in an attempt to establish educational programme protocols that suit different cohorts.

The study aimed to develop a health education programme targeting a miscellaneous group of Egyptian elderly in nursing homes to improve awareness regarding medications, with the ultimate goal of improving adherence and decreasing complications.

Reviewing the data collected regarding medications, the study revealed that 77.8% of the elderly at the nursing homes studied took their medication without assistance. A total of 70% of the elderly keep their medications in their own rooms in different arrangements, e.g., a medication bag, medication drawer, or a designated spot. A total of 67% of the elderly check or ask someone to check the expiration date on their medications. Almost half of the patients (46.5%) ask their physician before refilling their medications, while 35% refill by themselves. From the above data, it seems that despite living at a nursing home, the elderly take responsibility for their own medications. This finding magnifies the importance of educating them about storage, intake, refill, and other important aspects of their medications.

Focusing on the pre- and postintervention (educational programme) results from the three questionnaires used, we found the following.

The MedMaIDE questionnaire showed no significant statistical difference regarding nursing home participants' medication knowledge before and after intervention, while there were variable degrees of significant statistical differences in how the participants take their medications and obtain their medications and in their total deficiency scores before and after intervention. There was a highly significant decrease in the total deficiency score of medication management after health education: the nursing home participants' medication management actually improved, as has been similarly reported in many studies [25–27].

As for the MAQ, there was a significant statistical difference in participants' medication adherence after intervention: the original 34% of low-adherence participants dropped to only 18%, while the original 22% of high-adherence participants increased to 28% (*p* value 0.004). This result is consistent with the results of Mann et al. in a study published in 2019 that was conducted on older adults at risk for nonadherence in Houston, Texas, USA. These older adults were enrolled in a Medicare Advantage Plan with prescription drug coverage and received targeted mailing interventions to improve their adherence to medications for different chronic diseases, and 24.1% increase in adherence was detected. Similar results have been found in other studies that tested the effect of different models of health education on participants' adherence [28, 29].

There was no significant statistical difference in pre- and postintervention answers to the questions “Do you ever forget to take your medicine?” and “Are you careless at times about taking your medicine?” in the MAQ which could be due to the fact that 36.8% received help with taking medication, and 12.3% received reminders to take their medication. However, there was a highly significant statistical improvement in the response to the questions (increased “no” response) “When you feel better, do you sometimes stop taking your medicine?” and “Sometimes, if you feel worse when you take the medicine, do you stop taking it?” in the MAQ before and after intervention among nursing home participants. These results show that the educational program has improved medication adherence due to specific domains and not all. Medication adherence is multifactorial; patient-related factors being a primary domain, hence, “a key component of any adherence-improving plan is patient education.” [30]

Adherence was also positively correlated to specific-necessity beliefs. On the contrary, it was negatively correlated to specific-concern beliefs; participants with strong beliefs that their medications are necessary for their health will be better adherent and vice versa, which was in harmony with the finding of a cross-sectional interviewer-assisted survey conducted on patients with chronic diseases taking medications over one-year duration at family medicine clinics in Saudi Arabia to find the relationship between adherence and beliefs about medications [31]. Moreover, another cross-sectional study done in two large teaching hospitals in China to investigate beliefs about medicines and their association with medicine adherence in patients with chronic diseases like diabetes and rheumatoid arthritis concluded the same correlation [32]. Many other studies confirmed the same findings in the current study [33–35]. On the contrary, in a study done in Edo State, Nigeria, to determine the correlation between medication beliefs and medication adherence among patients with diabetes, it showed negative correlation between patients' adherence and their beliefs. That study was conducted in a single state with a culture that does not acknowledge modern medicine and prefers traditional medicine. Moreover, the sample of that study was from a single centre [36].

Health education did not change participants' beliefs about medications; the statistical analysis revealed no change in scores in mean or standard deviation before intervention and after intervention among nursing home participants (34.39 ± 6.196, 34.57 ± 6.220, respectively). This might be due to the high preintervention scores on beliefs.

In conclusion, the health education programme has improved medication management and adherence but not beliefs. We advise that the health programme be routinely used in nursing homes. We also recommend further studies to investigate whether similar results can be produced in the community-dwelling elderly in the Egyptian community and other similar cultures. It is also advisable to design an educational programme for health professionals and nursing home staff for nonpatient factors that affect medication management and adherence.

Disease-specific educational programmes such as the study by Shah and colleagues should also be developed for more specific targets. This study concluded that pharmacist-led educational interventions can be effective in promoting medication adherence and can be a good solution to improving the medication adherence of patients with heart failure. Healthcare providers should continue to provide education to patients about the importance of taking medications as prescribed [37].

### 4.1. Limitations

There were difficulties in getting the approval of the administration of many nursing homes (either governmental or private) to participate in the study even after knowing that this study is not related to any governmental institution or any media. This limited the diversity of nursing homes included.

## Figures and Tables

**Table 1:** How the elderly keep their medications in a sample of 200 nursing home Egyptian elderly residents (2017–2019).

Items	Number	Percentage (%)
The patient keeps his medications in the refrigerator	16	8
The patient keeps his medications in a special drawer for medicine	67	33.5
The patient keeps his medications in a bag for medicine	56	28
The patient keeps his medications in a pill box	8	4
The patient does not have a special place for medications	53	26.5
Medications are kept in the patient's own room	140	70
Medications are kept in the dining room	0	0
Medications are kept in the living room	35	17.5
Medications are kept in the kitchen	25	12.5
The patient checks the expiration date of medications or asks someone to check it	134	67
The patient does not check the expiration date of medications or asks someone to check it	66	33

**Table 2:** How the elderly obtain their medications in a sample of 200 nursing home Egyptian elderly residents (2017–2019).

Items	Number	Percentage (%)
The patient does not refill his prescription	15	7.5
The patient refills his prescription on his own	70	35
The patient contacts the doctor to refill his prescription	93	46.5
The patient contacts the pharmacist to refill his prescription	22	11
The patient makes sure the medications refilled are the same name as the ones finished	138	69
The patient does not make sure the medications refilled are the same name as the ones finished	62	31
The patient keeps his prescriptions	93	46.5
The patient does not keep his prescriptions	107	53.5
The patient needs medication that he/she cannot obtain due to lack of money	39	41.9
The patient needs medication that he/she cannot obtain due to shortage of that medication in the market	54	58.1

**Table 3:** Comparison of MedMaIDE score items among 50 Egyptian elderly nursing home residents before and after intervention (educational program 2017–2019).

Item	Mean and std deviation				*p* value
1. Persons do not know their medications (score)	Preintervention	0.9 ± 0.707	Postintervention	0.82 ± 0.661	569
2. Persons do not know how to take their medications (score)	Preintervention	0.58 ± 0.091	Postintervention	0.46 ± 0.082	**032** ^*∗*^
3. Persons do not know how to obtain their medications (score)	Preintervention	0.72 ± 0.095	Postintervention	0.5 ± 0.091	**002** ^*∗*^
4. Medication management deficiency total score	Preintervention	2.18 ± 0.178	Postintervention	1.78 ± 0.17	**000** ^*∗*^

^*∗*^ *p* value was calculated using the paired *t*-test.

**Table 4:** Comparison between MAQ categories among 50 Egyptian elderly nursing home residents before and after intervention (educational program 2017–2019).

	Preintervention		Postintervention		*p* value
Low adherence	17	34%	9	18%	**0.004** ^*∗*^
Medium adherence	22	44%	27	54%	
High adherence	11	22%	14	28%	

^*∗*^ *p* value was calculated using the McNemar test.

**Table 5:** Comparison between MAQ items among 50 Egyptian elderly nursing home residents before and after intervention (educational program 2017–2019).

	Postintervention	Preintervention		*p* value
		Yes	No	
1-Do you ever forget to take your medicine?	Yes	19 (38%)	0 (0%)	1.000
	No	0 (0%)	31 (62%)	

2-Are you careless at times about taking your medicine?	Yes	21 (42%)	0 (0%)	1.000
	No	0 (0%)	29 (58%)	

3-When you feel better, do you sometimes stop taking your medicine?	Yes	15 (30%)	0 (0%)	**0.000** ^*∗*^
	No	14 (28%)	21 (42%)	

4-Sometimes, if you feel worse when you take the medicine, do you stop taking it?	Yes	7 (14%)	0 (0%)	**0.000** ^*∗*^
	No	16 (32%)	27 (54%)	

^*∗*^ *p* value was calculated using McNemar test.

## Data Availability

The data used to support the findings of this study are available from the corresponding author upon request.
